# Case report: Invasive *Klebsiella pneumoniae* liver abscess syndrome

**DOI:** 10.3389/fmed.2025.1643389

**Published:** 2025-08-14

**Authors:** Yiwei Wang, Miao Dai, Meixian Lei

**Affiliations:** ^1^Jiujiang CityKey Laboratory of Cell Therapy, Department of Infectious Diseases, Jiujiang No.1 People’s Hospital, Jiujiang, Jiangxi, China; ^2^Department of Geriatrics, Chronic Disease Management Center, Jiujiang No.1 People’s Hospital, Jiujiang, Jiangxi, China; ^3^Department of Cardiology, Jiujiang No.1 People’s Hospital, Jiujiang, Jiangxi, China

**Keywords:** *Klebsiella pneumoniae*, liver abscess, septic pulmonary embolism, invasive infection, antimicrobial therapy

## Abstract

Invasive *Klebsiella pneumoniae* liver abscess syndrome (IKLAS) is a severe condition characterized by liver abscesses with systemic complications, often caused by hypervirulent strains. We present a 65-year-old woman with no predisposing factors who developed IKLAS complicated by septic pulmonary embolism and polymicrobial co-infections. The patient presented with a one-month history of right upper abdominal pain, fever (39.5°C), and respiratory distress. Initial laboratory findings revealed leukocytosis (WBC 15.97 G/L), elevated inflammatory markers (CRP 83.04 mg/dL, PCT 96.9 ng/mL), and hepatic dysfunction (ALT 361.7 U/L, AST 573.9 U/L). Imaging identified a massive liver abscess (153.7 × 112.6 mm) and septic pulmonary emboli. Blood and pus cultures confirmed *Klebsiella pneumoniae* (susceptible to imipenem/cefoperazone-sulbactam), prompting targeted therapy. Despite initial drainage and antibiotics, her condition deteriorated due to secondary hospital-acquired infections with *Acinetobacter baumannii*-calcoaceticus complex and Pichia ommerica, necessitating escalation to meropenem and voriconazole. This adjustment led to clinical resolution, with abscess reduction to 74.0 × 46.0 mm on follow-up imaging and normalization of laboratory parameters. The patient was discharged after completing antimicrobial therapy. This case underscores three critical lessons: IKLAS requires high suspicion in atypical presentations (e.g., isolated abdominal pain), as delays risk metastatic complications; polymicrobial infections may emerge secondary to invasive procedures, necessitating repeated microbiological evaluation; and large abscesses (>100 mm) often demand prolonged, tailored therapy and multidisciplinary management. Our findings highlight the importance of early imaging, comprehensive pathogen identification, and adaptive treatment strategies to improve outcomes in this complex syndrome.

## Introduction

Invasive *Klebsiella pneumoniae* liver abscess syndrome (IKLAS) is a severe clinical entity characterized by liver abscesses associated with metastatic complications such as septic pulmonary embolism (1), endophthalmitis (2), meningitis (3, 4), and spondylodiscitis (5). It is predominantly caused by hypervirulent strains of *Klebsiella pneumoniae*, which often affect otherwise healthy individuals. The incidence of IKLAS is increasing, particularly in Asia, highlighting the need for improved diagnostic and therapeutic strategies (6).

Epidemiological studies show that hvKP strains are responsible for a significant proportion of pyogenic liver abscesses, with reported rates of metastatic complications ranging from 12 to 28% (7, 8). Diagnosis remains challenging due to nonspecific early symptoms, and imaging studies, especially CT, are crucial for early detection. Blood cultures and abscess drainage are essential for microbiological confirmation, though polymicrobial infections may complicate management. Treatment typically involves antimicrobial therapy guided by culture and sensitivity results, with percutaneous drainage or surgical intervention for large abscesses.

This case report highlights the diagnostic and therapeutic challenges of IKLAS, emphasizing the importance of early suspicion, comprehensive microbiological assessment, and adaptive treatment strategies in improving outcomes.

## Case report

A 65-year-old woman with no significant medical history-including no diabetes, chronic liver disease, immunocompromising conditions, or recent invasive procedures-presented with a one-month history of progressive right upper quadrant (RUQ) abdominal pain exacerbated by deep inspiration and bending. She denied recent travel, freshwater exposure, or similar prior episodes. On admission, her vital signs were notable for fever (39.5°C), tachycardia (149 bpm), tachypnea (31 breaths/min), and hypertension (159/87 mmHg). She exhibited altered mental status and respiratory distress. Physical examination revealed RUQ tenderness without rebound or guarding. No hepatomegaly, Murphy’s sign, or palpable masses were detected.

**Figure 1:**
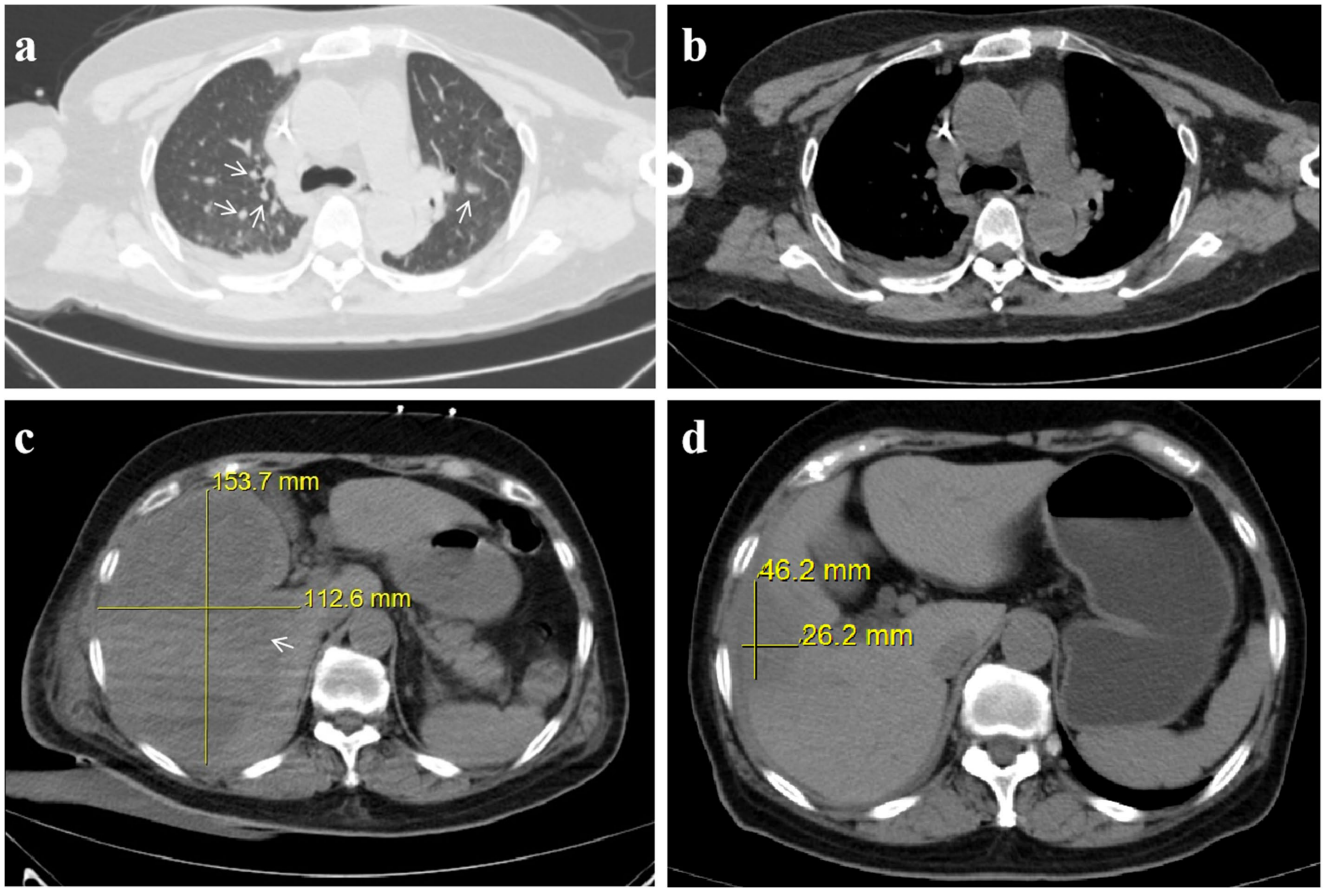
Invasive hepatic abscess syndrome with septic pulmonary embolism caused by *Klebsiella pneumoniae*: chest and abdominal CT. **(a)** Chest CT in the lung window showing scattered nodules (arrow). **(b)** Chest CT in the mediastinal window. **(c)** Pre-treatment abdominal CT reveals a 153.7 × 112.6 mm liver abscess. **(d)** Post-treatment abdominal CT reveals a 46.2 × 26.2 mm liver abscess.

On July 12, 2024, laboratory tests revealed leukocytosis (white blood cell count 16.0 × 10^9^/L), anemia (hemoglobin 98.0 g/L), and markedly elevated inflammatory markers (C-reactive protein 83.0 mg/dL; procalcitonin 96.9 ng/mL). Liver function tests indicated significant hepatocellular injury (alanine aminotransferase 361.7 U/L, aspartate aminotransferase 573.9 U/L), while renal impairment was evidenced by elevated serum creatinine (190.3 μmol/L) and urea (15.8 mmol/L). Coagulopathy was suggested by a high plasma D-dimer level (32.3 mg/L) (Table 1). Chest computed tomography (CT) demonstrated scattered nodular opacities and patchy consolidations in both upper lobes, consistent with septic pulmonary embolism, along with a small right pleural effusion (Figures 1a,b). Abdominal CT revealed a large hypodense lesion (153.7 × 112.6 mm) in the right hepatic lobe, suggestive of a liver abscess (Figure 1c). *Klebsiella pneumoniae* was isolated from both percutaneous hepatic abscess drainage and blood cultures. Antimicrobial susceptibility testing (performed per Clinical and Laboratory Standards Institute [CLSI] M100, 33rd edition [2023] guidelines) confirmed susceptibility to imipenem and cefoperazone-sulbactam (Table 2) (9). Minimum inhibitory concentration (MIC) breakpoints and interpretations followed CLSI criteria.

**Table 1:** Clinical and laboratory variables.

Blood analytes	July 12th, 2024	July 16th, 2024	July 20th, 2024	July 25th, 2024	August 21th, 2024	Normal value
White blood cell	16.0	11.5	8.6	11.1	6.7	3.5–9.5 × 10^9^/L
Neutrophil	14.4	9.9	6.8	9.3	3.2	1.8–6.3 × 10^9^/L
Hemoglobin	98.0	89.0	82.0	82.0	99.0	130–175 g/L
Platelets	91.0	152.0	473.0	456.0	371.0	125–350 × 10^9^/L
C-reactive protein	83.0	82.0	77.2	83.7	0.7	0–1.0 mg/dL
PCT	96.9	12.2	2.4	95.7	0.1	0–0.25 ng/mL
IL-6	482.0	90.5	95.3	_	_	0-7 pg./mL
Alanine aminotransferase	361.7	111.0	86.0	72.0	24.0	0-42 U/L
Aspartate aminotransferase	573.9	87.0	81.0	78.0	23.0	0-40 U/L
D-dimer	32.3	29.7	30.2	30.8	2.6	0–1.0 μg/mL

**Table 2:** Susceptibility test of *Klebsiella pneumoniae* isolated from the blood and pus cultures.

Antibiotic	MIC (μg/mL)	Susceptibility
Amikacin	≤2	S
Aztreonam	≤1	S
Ciprofloxacin	≤0.25	S
Colistin	≤0.5	S
Doxycycline	1	S
Cefepime	≤0.12	S
Imipenem	≤0.25	S
Levofloxacin	≤0.12	S
Meropenem	≤0.25	S
Minocycline	4	S
Cefoperazone/sulbactam	≤8	S
Cotrimoxazole	≤20	S
Ceftazidime	≤0.12	S
Ticacillin/clavulanic acid	≤8	S
Tigecycline	≤0.5	S
Tobramycin	≤1	S
Piperacillin/tazobactam	≤4	S

MIC, minimum inhibitory concentration; S, Susceptible.

Initial antimicrobial therapy was guided by *Klebsiella pneumoniae* isolated from both hepatic abscess pus and blood cultures, which demonstrated susceptibility to imipenem and cefoperazone-sulbactam (Table 2). The patient received intravenous imipenem (1.0 g every 8 h) for 8 days, followed by de-escalation to cefoperazone-sulbactam (3.0 g every 8 h) on July 20, 2024, for an additional 5 days. Supportive measures included high-flow oxygen therapy, prophylactic nadroparin calcium, and nutritional optimization. Despite these interventions, the patient exhibited persistent symptoms, including recurrent fever (peak temperature: 39.5°C), ongoing abdominal pain, and worsening respiratory distress. Repeat abdominal ultrasound revealed an enlarged hepatic abscess with septations and minimal liquefaction, suggesting inadequate response to both percutaneous drainage and initial antibiotic therapy. Microbiological analysis of catheter secretions and blood cultures revealed the presence of additional pathogens: the *Acinetobacter baumannii*-calcoaceticus complex (susceptible to meropenem and tigecycline) and Pichia ommerica (susceptible to voriconazole). These pathogens were likely acquired nosocomially, given their detection after invasive interventions and prolonged catheter use. These findings highlighted that the initial antimicrobial therapy was not targeting all causative organisms, and the ongoing systemic infection was evidenced by elevated inflammatory markers (CRP 83.7 mg/dL, PCT 95.7 ng/mL) by July 25th, 2024 (13 days post-treatment initiation) (Table 1). Based on the clinical and microbiological findings (Tables 3, 4), the antimicrobial therapy was escalated to a combination of meropenem (1.0 g every 8 h), tigecycline (initial dose of 100 mg, followed by 50 mg every 12 h via intravenous infusion for maintenance), and voriconazole for antifungal coverage. Voriconazole was administered intravenously (6 mg/kg every 12 h for the first 24 h, followed by 4 mg/kg every 12 h as maintenance) throughout the treatment course to maintain therapeutic levels, given the patient’s severe infection and clinical instability. The combined anti-infective regimen was maintained for 14 days. This combination was specifically chosen to target the newly identified pathogens, ensuring broader antimicrobial coverage and addressing the polymicrobial nature of the infection.

**Table 3:** Susceptibility test of *Acinetobacter baumannii*-calcoaceticus complex isolated from central venous catheter secretion cultures.

Antibiotic	MIC (μg/mL)	Susceptibility
Amikacin	6	R
Ceftazidime	6	R
Ciprofloxacin	6	R
Cefoperazone/sulbactam	10	R
Cefepime	6	R
Gentamicin	6	R
Imipenem	16	R
Levofloxacin	11	R
Meropenem	≤4	S
Minocycline	11	I
Piperacillin	6	R
Ampicillin/sulbactam	6	R
Selectrin	6	R
Tigecycline	17	S
Tobramycin	6	R
Piperacillin/tazobactam	6	R

MIC, minimum inhibitory concentration; S, Susceptible; I, Intermediate; R, Resistance.

**Table 4:** Susceptibility test of Pichia ommerica isolated from central venous catheter blood cultures.

Antibiotic	MIC (μg/mL)	Susceptibility
Amphotericin B	≤0.5	S
5-fluorocytosine	≤4	S
fluconazole	2	S
Itraconazole	≤0.125	S
Voriconazole	≤0.06	S

MIC, minimum inhibitory concentration; S, Susceptible.

Following this adjustment, the patient’s condition improved significantly, with fever resolution, decreased abdominal pain, and stabilized clinical status. The drainage catheter stopped producing purulent fluid, and ultrasound showed a reduction in abscess size to 74.0 × 46.0 mm. Blood tests confirmed normalized inflammatory markers and liver and kidney function, allowing for the discontinuation of meropenem, tigecycline, and voriconazole on August 8th, 2024. The patient subsequently completed an additional 21-day course of cefoperazone-sulbactam (3.0 g every 8 h). Follow-up abdominal CT prior to treatment completion revealed a marked reduction in abscess dimensions to 46.2 × 26.2 mm (Figure 1d), with no residual purulent drainage on August 29th, 2024.

At a 3-month follow-up, the patient remained asymptomatic with no evidence of residual abscess or metastatic complications on repeat imaging. Liver function tests and inflammatory markers (CRP < 5 mg/dL, PCT < 0.05 ng/mL) were within normal limits, confirming full biochemical recovery. She denied persistent pain or respiratory symptoms and scored 85/100 on the SF-36 quality-of-life questionnaire, indicating good functional recovery. No psychiatric sequelae were noted.

## Discussion

Invasive *Klebsiella pneumoniae* liver abscess syndrome (IKLAS) is a severe infection characterized by liver abscesses caused by presumptive hypervirulent *K. pneumoniae* (hvKP) strains. Unlike classical *K. pneumoniae*, hvKP exhibits enhanced virulence factors (e.g., hypermucoviscosity, rmpA genes) and frequently infects healthy individuals without predisposing conditions (10). The epidemiology of IKLAS has garnered significant attention, particularly due to its increasing prevalence in East and Southeast Asia. The incidence of hvKP-related liver abscesses has been rising, with a notable proportion of cases developing metastatic infections such as septic pulmonary embolism and endophthalmitis (1, 7). Over the last 30–40 years, *Klebsiella pneumoniae* has replaced *Escherichia coli* as the dominant and overwhelming pathogen (11). Additionally, the incidence of endophthalmitis in patients with hvKP liver abscess ranges from 3.4 to 12.6%, highlighting the invasive nature of these strains (12). This case of a 65-year-old woman with a massive liver abscess measuring 153.7 × 112.6 mm is particularly notable for its size, complexity, and the coexistence of secondary infections, highlighting the diagnostic and therapeutic challenges of IKLAS.

IKLAS is increasingly recognized for its heterogenous presentations, ranging from indolent abdominal pain to fulminant sepsis. Unlike classical pyogenic liver abscesses, hvKP-associated IKLAS often occurs in immunocompetent hosts, as seen in our patient, who lacked traditional risk factors like diabetes or biliary disease (13). Although capsular typing was not performed in this case, the rapid clinical progression and metastatic spread strongly suggest an hvKP strain. Among a large cohort of over 800 patients with *K. pneumoniae* liver abscesses across Taiwan, South Korea, and the United States, metastatic disease was observed in 12% of cases (7), though reported rates vary and may reach as high as 28% (8). The most frequent metastatic sites include the eye, central nervous system, and lung (14–17). Our patient’s massive abscess (153.7 × 112.6 mm) and septic pulmonary embolism exemplify the severe end of the spectrum, reinforcing the need for early imaging in unexplained fever or abdominal pain, even without classic risk factors. Studies suggest that CT should be prioritized over ultrasound due to higher sensitivity for small abscesses and metastatic foci (18, 19). The diagnostic challenge lies in the nonspecific early symptoms. Our patient’s month-long abdominal pain without initial fever highlights the pitfalls of delayed imaging. Blood cultures and abscess drainage remain cornerstones for microbiological confirmation, but polymicrobial infections (e.g., *Acinetobacter*, *Pichia* spp.) may emerge later, necessitating repeated sampling (20). IKLAS is predominantly caused by hvKP strains, which are often monomicrobial in origin. However, as seen in our case, polymicrobial infections (e.g., *Acinetobacter baumannii*-calcoaceticus complex and Pichia ommerica) may arise secondary to prolonged hospitalization or invasive procedures, complicating management. This case underscores the need for vigilance against secondary pathogens in refractory IKLAS, particularly in catheter-dependent patients.

The initial antimicrobial regimen of imipenem and cefoperazone-sulbactam was selected based on the susceptibility profile of *Klebsiella pneumoniae* isolated from blood and abscess cultures (Table 2), aligning with Chinese Expert Consensus for pyogenic liver abscesses (e.g., carbapenems as first-line for severe infections) (21). However, the patient’s persistent fever, rising inflammatory markers, and imaging showing abscess progression (septation, minimal liquefaction) prompted reevaluation. Repeat cultures identified *Acinetobacter baumannii*-calcoaceticus complex (resistant to imipenem but susceptible to meropenem/tigecycline) and Pichia ommerica (susceptible to voriconazole), indicating polymicrobial infection likely secondary to catheter colonization.

Escalation to meropenem (broadened coverage for Acinetobacter), tigecycline (for multidrug-resistant Gram-negative pathogens), and voriconazole (targeting fungemia) was guided by antimicrobial susceptibility test results (Tables 2, 3) and clinical failure criteria per IDSA recommendations. The 21-day de-escalation to cefoperazone-sulbactam was justified by resolution of systemic symptoms, normalized labs, and imaging showing abscess reduction (74.0 × 46.0 mm), ensuring targeted therapy while minimizing resistance risk. This adaptive approach underscores the importance of serial microbiological monitoring and guideline-based adjustments in polymicrobial IKLAS.

The pathogenesis of IKLAS is thought to involve bacterial virulence factors such as the production of siderophores, capsule formation, and the expression of regulators of mucoid phenotype A (rmpA) genes, which enhance bacterial survival and dissemination (11). The absence of classical risk factors in this patient raises the possibility of host-specific immune deficiencies or undiagnosed conditions that predispose to hvKP infections. Further studies are needed to elucidate whether hypervirulent strains exhibit enhanced abilities to evade immune surveillance in otherwise healthy hosts. The subsequent detection of *A. baumannii* and P. ommerica in catheter secretions and blood cultures underscores the risk of secondary nosocomial infections in prolonged hospitalizations. These were not part of the initial polymicrobial infection but arose due to invasive procedures and biofilm formation on indwelling catheters. *Acinetobacter baumannii*-calcoaceticus complex, a biofilm-forming pathogen, likely colonized the drainage catheter, complicating source control. The Acinetobacter isolate’s resistance to imipenem but susceptibility to meropenem suggests possible carbapenemase production or efflux pump activity, common in *A. baumannii*. This underscores the need for AST-guided therapy in polymicrobial infections. Meropenem’s efficacy may reflect its stability against certain carbapenemases or lower affinity for efflux pumps, explaining the clinical improvement post-escalation. Concurrent Pichia ommerica fungemia further amplified systemic inflammation, necessitating dual antimicrobial therapy. This highlights the imperative for rigorous catheter care and serial microbiological surveillance in IKLAS management.

This case underscores critical considerations for clinicians managing IKLAS, particularly in atypical or refractory presentations. First, atypical manifestations (e.g., isolated abdominal pain without fever) necessitate early CT imaging and microbiological testing to avoid delayed diagnosis and metastatic complications like septic embolism. Second, serial microbiological evaluation is essential, as initial cultures often miss polymicrobial infections (e.g., secondary *Acinetobacter* and *Pichia* spp. here), requiring therapy escalation. The efficacy of meropenem against carbapenem-resistant Acinetobacter and voriconazole for fungal co-infections highlights the need for tailored regimens in such scenarios. Third, large abscesses (>100 mm) frequently respond poorly to initial drainage; early adjunctive measures (catheter irrigation, prolonged targeted therapy) should be considered. Fourth, invasive procedures risk nosocomial infections, emphasizing the importance of strict catheter care and antifungal prophylaxis in refractory cases. Aggressive source control must be balanced with vigilance for iatrogenic complications. The prolonged broad-spectrum therapy in this case was justified by polymicrobial etiology and delayed response, though future studies should clarify optimal duration and de-escalation strategies. Notably, the patient’s lack of traditional risk factors invites further research into host-pathogen interactions in hypervirulent *K. pneumoniae* infections.

This study has several limitations. First, molecular and phenotypic characterization of the *Klebsiella pneumoniae* isolate (e.g., capsular serotyping, hypermucoviscosity testing, or rmpA gene detection) was not performed, which could have confirmed its hypervirulent strain status and elucidated pathogen-specific virulence mechanisms. Second, carbapenemase testing for the *Acinetobacter baumannii*-calcoaceticus complex was not conducted, leaving the exact resistance mechanism (e.g., carbapenemase production vs. efflux pump activity) unresolved. Third, while the infection was classified as polymicrobial based on microbiological findings, the secondary pathogens (*A. baumannii* and Pichia ommerica) likely represented nosocomial acquisitions from prolonged hospitalization and invasive procedures rather than true co-pathogens in the initial abscess. Future studies should incorporate advanced molecular diagnostics to refine pathogen characterization and resistance profiling in such complex cases.

This case highlights the complexity of managing massive IKLAS, particularly in the context of secondary infections. While initial management aligns with established guidelines, the unique challenges posed by secondary infections and biofilm-associated pathogens necessitate a more nuanced, multidisciplinary approach. The findings emphasize the importance of serial evaluations, pathogen-specific therapy, and close monitoring in ensuring favorable outcomes for patients with IKLAS.

## Data Availability

The raw data supporting the conclusions of this article will be made available by the authors, without undue reservation.
